# Alkaloids from *Cryptolepis sanguinolenta* as Potential Inhibitors of SARS-CoV-2 Viral Proteins: An *In Silico* Study

**DOI:** 10.1155/2020/5324560

**Published:** 2020-09-22

**Authors:** Lawrence Sheringham Borquaye, Edward Ntim Gasu, Gilbert Boadu Ampomah, Lois Kwane Kyei, Margaret Amerley Amarh, Caleb Nketia Mensah, Daniel Nartey, Michael Commodore, Abigail Kusiwaa Adomako, Philipina Acheampong, Jehoshaphat Oppong Mensah, David Batsa Mormor, Caleb Impraim Aboagye

**Affiliations:** ^1^Department of Chemistry, Kwame Nkrumah University of Science and Technology, Kumasi, Ghana; ^2^Central Laboratory, Kwame Nkrumah University of Science and Technology, Kumasi, Ghana

## Abstract

The ongoing global pandemic caused by the human coronavirus, severe acute respiratory syndrome coronavirus 2 (SARS-CoV-2), has infected millions of people and claimed hundreds of thousands of lives. The absence of approved therapeutics to combat this disease threatens the health of all persons on earth and could cause catastrophic damage to society. New drugs are therefore urgently required to bring relief to people everywhere. In addition to repurposing existing drugs, natural products provide an interesting alternative due to their widespread use in all cultures of the world. In this study, alkaloids from *Cryptolepis sanguinolenta* have been investigated for their ability to inhibit two of the main proteins in SARS-CoV-2, the main protease and the RNA-dependent RNA polymerase, using *in silico* methods. Molecular docking was used to assess binding potential of the alkaloids to the viral proteins whereas molecular dynamics was used to evaluate stability of the binding event. The results of the study indicate that all 13 alkaloids bind strongly to the main protease and RNA-dependent RNA polymerase with binding energies ranging from -6.7 to -10.6 kcal/mol. In particular, cryptomisrine, cryptospirolepine, cryptoquindoline, and biscryptolepine exhibited very strong inhibitory potential towards both proteins. Results from the molecular dynamics study revealed that a stable protein-ligand complex is formed upon binding. Alkaloids from *Cryptolepis sanguinolenta* therefore represent a promising class of compounds that could serve as lead compounds in the search for a cure for the corona virus disease.

## 1. Introduction

The novel human coronavirus was reported in China in late 2019. Ever since, the virus has spread throughout the world and has been designated a pandemic [1]. This novel human coronavirus has been named as severe acute respiratory syndrome coronavirus 2 (SARS-CoV-2), and the disease it causes is also called the corona virus disease 2019 (COVID-19) [2, 3]. As at May 21st, 2020, over 5 million cases have been recorded globally and the number of confirmed deaths is in excess of 300,000. The impact of the disease has been felt in all spheres of life, with devastating effects on the healthcare, social, and economic fabric of many countries.

As a ribonucleic acid (RNA) virus, SARS-CoV-2 is similar to the viruses that caused the Middle East respiratory syndrome (MERS) and the severe acute respiratory syndrome (SARS). In terms of biological classification, coronaviruses belong to the order Nidovirales and the family Coronaviridae [4]. There are four genera of the coronaviruses, namely, alpha-, beta-, gamma-, and delta-coronavirus [5]. SARS and MERS are both in the genus *beta-coronavirus*. These coronaviruses possess single strands of RNA and are enveloped in a protein with spike-like structures that project out of the surface of the envelope. SARS-CoV-2 uses angiotensin-converting enzyme 2 (ACE2) as a receptor during infection [6]. Infection, persistence, pathogenesis, and reemergence after viral maturation in hosts are mostly regulated by essential viral proteins.

Due to the crucial role they play in replication of a virus, proteases are usually earmarked as key targets in antiviral drug development [7]. The genome of SARS-CoV-2 is made up of about 30,000 nucleotides, and this RNA encodes polyproteins required for replication and transcription of the virus. When translated, the polyproteins are extensively processed via proteolysis. The main protease (M^pro^) of SARS-CoV-2 processes the polypeptides by cleaving them at about 11 conserved locations to yield functional proteins. The functional 33.8 kDa M^pro^ is a symmetrical dimer, with each unit made up of 3 domains. The first 2 domains (I and II) possess an antiparallel *β*-barrel structure whereas the third domain (III) is made up of 5 *α*-helices arranged into an antiparallel globular knot. Domain II is joined to domain III via an extended loop region. A Cys-His catalytic dyad is at the heart of the catalytic activities of the SARS-CoV-2 M^pro^, and the substrate binding pocket in the protease can be found in a cleft situated between domains I and II [8, 9]. Humans lack closely homologous proteins to M^pro^, and this makes the SARS-CoV-2 M^pro^ a potentially important target in antiviral therapy development [10].

The SARS-CoV-2 RNA-dependent RNA polymerase is also one attractive drug target. The RNA-dependent RNA polymerase utilizes metal cofactors to catalyze the formation of phosphodiester bonds between 2 ribonucleotides during RNA replication. The structure of the polymerase is characterized by the presence of 2 important domains: a polymerase or RdRp domain and a nidovirus RdRp nucleotidyltransferase (NiRAN) domain. Connecting these 2 is an interface domain. There is an N-terminal *β*-hairpin structure that is found as an insert in a groove clamped on either side by the NiRAN domain and the RdRp domain. The conserved polymerase motifs A-G in the palm domain of the RdRp constitute the active site pocket of the SARS-CoV-2 RNA-dependent RNA polymerase [11]. The points of entry of the RNA template or primer and the nucleoside triphosphates, as well as the exit point of the emerging RNA strand, are all positively charged and solvent accessible. These points of entry, composed of motifs A and C through motifs F and G supported by E and the thumb, and exit points (the RNA exit tunnel), converge in a central cavity where template-directed RNA synthesis is carried out [12].

The rapid global spread of COVID-19 has underscored the need for the development of potent anti-COVID-19 therapeutics to combat this pandemic. Lessons from SARS and MERS, clinical drug repurposing, and *in vitro* high throughput screening have inspired novel insights towards the discovery of anti-COVID-19 drug candidates [13]. While these have suggested a few old drugs (such as remdesivir, lopinavir, hydroxychloroquine, and their azithromycin combinations) with new tricks against COVID-19, experimental techniques have also proposed macromolecular targets for attenuating viral replication [9, 12]. The presence of high resolution structures of important viral proteins provides an avenue for their use *in silico* techniques such as molecular docking and molecular dynamics simulations to screen and evaluate potential inhibitors [7].

According to the World Health Organization (WHO), 65-80% of the world's population depends on herbal medicine in treating various diseases [14]. Herbal preparations and medicinal plants represent a potential source of therapeutics in this time of great need for antiviral agents that can help in fighting COVID-19. *Cryptolepis sanguinolenta* is a widely used plant in West African herbal medical practice. Extracts from the plant are used in treating ailments like diabetes, hypertension, malaria, respiratory diseases, and diarrhea [15–19]. Several alkaloids have been isolated from the plant, and these compounds and the plant extracts possess broad spectrum antipathogenic activity [17, 18, 20, 21]. The plant extract is also used in managing hepatitis B viral infection and liver damage [18, 22]. Available data from the literature suggests the effectiveness of *Cryptolepis sanguinolenta* extracts in interfering with viral replication of the herpes simplex virus type 1 [22]. The extensive use of the plant in folkloric viral therapy and the indication that it interferes with viral replication motivated us to evaluate compounds isolated from the plant as potential inhibitors of SARS-CoV-2 viral proteins.

This work examined alkaloids from *Cryptolepis sanguinolenta* as potential inhibitors of the SARS-CoV-2 main protease and RNA-dependent RNA polymerase using *in silico* techniques. The isolated alkaloids examined are quindoline, cryptospirolepine, cryptolepine, hydroxycryptolepine, neocryptolepine, cryptomisrine, cryptolepicarboline, 11-isopropylcryptolepine, cryptolepinone, biscryptolepine, isocryptolepine, cryptoheptine, and cryptoquindoline [17]. Molecular docking was used to estimate binding affinities of the alkaloids towards the proteins and determine important interactions that mediate binding whereas molecular dynamics simulations were used to assess stability of protein-ligand complexes. We herein report that the alkaloids of *Cryptolepis sanguinolenta* showed strong inhibitory potentials towards both the main protease and the RNA-dependent RNA polymerase and the association exhibited remarkable stability.

## 2. Methods

### 2.1. Target Proteins

#### 2.1.1. Main Protease (M^pro^)

The X-ray crystal structure of SARS-CoV-2 main protease (M^pro^) was obtained from the protein data bank (PDB ID: 6LU7) as a protein co-crystallized with a known peptide-like inhibitor, N3 (N-[(5-methylisoxazol-3-yl)carbonyl]alanyl-l-valyl-N~1~((1R,2Z)-4-(benzyloxy)-4-oxo-1-{[(3R)-2-oxopyrrolidin-3-yl]methyl}but-2-enyl)-L-leucinamide)[8]. N3 was bound to a pocket assumed to be the active site. The active site residues were obtained from the PDBSUM [23] entry for 6LU7 with binding site residues Thr24, Thr25, Thr26, His41, Phe140, Leu141, Asn142, Gly143, Ser144, Cys145, His163, His164, Met165, Glu166, Pro168, His172, Arg188, Gln189, and Thr190 for inhibitor N3. These residues were also confirmed in Discovery Studio 2017 R2 client (Dassault Systèmes BIOVIA (Discovery Studio Visualizer) (2017 R2 Client), San Diego: Dassault Systèmes (2017)). Important regions in the active site of M^pro^ were labelled with their original subdomain classification S1 (Phe140, Asp142, Glu166, His163, and His172), S1′ (Thr25, Thr26, His164, and Cys145), S2 (side chains of His41, Met49, Thr57, and Met165 as well as Asp189), and S4 (Met165, Leu167, Phe185, and Gln192) for simplicity.

#### 2.1.2. RNA-Dependent RNA Polymerase

Homology model of the RNA-dependent RNA polymerase of SARS-CoV-2 was accessed from the SWISS-MODEL web server with RefSeq ID YP_009725307.1 [24, 25]. A single particle electron microscopy structure of the RNA-dependent RNA polymerase was also obtained from the protein data bank (PDB ID: 6M71, resolution: 2.9 Å) [26]. Missing loops in 6M71 were replaced using MODELLER [27]. The homology model of the RNA-dependent RNA polymerase is designated RdRp whereas the electron microscopy structure is designated as RdRpol. Active site analysis was made and optimized in Discovery Studio. Grid dimensions of predicted sites were selected to cover motifs A to G as described elsewhere [12].

### 2.2. Molecular Docking and Visualization

#### 2.2.1. Protein Preparation

The proteins were prepared by removing all complexed ligands and maintaining water molecules, especially those in the active sites where applicable. Replacement of incomplete side chains was done using the default Dunbrack rotamer library. Protonation states were assigned for histidine while addition of polar hydrogen atoms to correct the calculation of partial charges for standard residues was also done. Rotamer and backbone secondary structure preferences were improved with AMBER ff14SB, and then Gasteiger charges were computed for each atom using Antechamber implemented in Chimera [28, 29].

#### 2.2.2. Ligand Preparation

**Figure 1:**
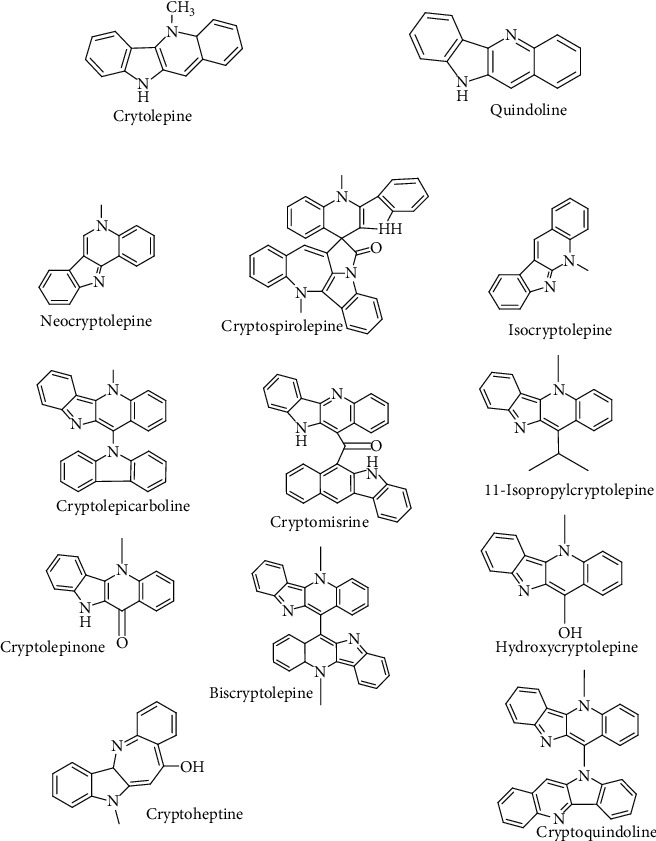
Structures of *Cryptolepis sanguinolenta* alkaloids used in this study.

A total of thirteen (13) alkaloids isolated from *Cryptolepis sanguinolenta* (Figure 1) were used for the docking studies. The ligands were sketched in Spartan'14 (Wavefunction Inc., Irvine California, USA) ChemDraw interface, modelled in 3D followed by SYBYL force field minimization as well as geometry optimization by equilibrium geometry estimation with the density functional theory (DFT) B3LYP/6-31G∗ basis set in vacuum. They were converted into pdb formats and prepared for docking by addition of polar hydrogens and Gasteiger charges. Additionally, curcumin, luteolin-7-glycoside, hydroxychloroquine, adenosine triphosphate (ATP), remdesivir, lopinavir, and nelfinavir were treated as above for validation purposes.

#### 2.2.3. Molecular Docking

Precision docking protocol was developed by docking the bound ligand into the prepared active sites. In order to verify that the pose resulting from *in silico* docking represent correctly bound conformations, it was visually inspected and compared to the experimentally determined binding modes and conformations of N3 in 6LU7. The DINC 2.0 web server was used to dock N3 with M^pro^ using estimated grid dimensions provided by the software since N3 is peptidic in nature [30]. The presence of conserved binding pocket interactions between N3 and the active site residues of M^pro^ validated the docking protocol used in the study. For docking involving non-peptide ligands, AutoDock Vina was used. The molecular docking studies were performed on all 13 ligands against M^pro^ using the following grid dimensions: center (Å): *X*: -10.7647, *Y*: 12.5092, and *Z*: 68.969 and size (Å): *X*: 28, *Y*: 28, and *Z*: 28. For RdRp and RdRpol, docking of ligands with the proteins was performed using the following grid dimensions: center (Å): *X*: 132.394, *Y*: 137.038, and *Z*: 165.346 and size (Å): *X*: 41, *Y*: 41, and *Z*: 41 for RdRp and center (Å): *X*: 123.115, *Y*: 115.699, and *Z*: 132.978 and size (Å): *X*: 41, *Y*: 41, and *Z*: 41 for RdRpol.

In all cases, docking was performed in three technical runs. Free energies were obtained from runs with the most consistent poses. These free energies were used for the estimation of the ligand binding constant (*K*_*d*_) and ligand efficiency (Δ*g*) [31]. Protein-ligand complexes were analyzed in Discovery Studio for interactions with pocket atoms based on default settings in the software. Discovery Studio was used to visualize docked structures as well as the generation of 3D and 2D schematic representations of protein-ligand interactions.

### 2.3. Molecular Dynamics Simulation

Ligands with binding affinities between -8.5 and -11.0 kcal/mol were considered for molecular dynamics (MD) simulations. In this regard, cryptomisrine, cryptospirolepine, cryptoquindoline, and biscryptolepine were used for MD simulation studies of M^pro^ whereas cryptomisrine, cryptospirolepine, and cryptoquindoline were used in MD simulation studies of RdRp and RdRpol. The Ligand and Receptor Molecular Dynamics (LARMD) web server was utilized for all MD simulations. The default MD simulation method on the LARMD web server was modified to suit the purpose of this study. The explicit water model was used in the simulation. Where necessary, Na^+^ or Cl^−^ was added to obtain a neutral system. Trajectory analysis and free energy calculation protocols were used in the default mode as found on the LARMD web server [32].

Protein-ligand complexes were written from the best pose obtained from Vina. Ligand atoms were assigned AM1-BCC charges using the Antechamber module whereas the coordinate and topology files of the complex were constructed with the teLeap module, all in the AMBER16 package [33, 34]. The AMBER ff14SB force field [28] and GAFF (General AMBER Force Field) [29, 35] were used for amino acid residues and ligands, respectively. The molecules were solvated in an octahedral box of TIP3P water [36] extended at least 10 Å in each direction from the solute [36, 37]. Initially, all atoms were fixed except for water, ions, and hydrogens. Thereafter, only the backbone atoms of the protein were fixed. Finally, the residues around the ligand within 6 Å were minimized, and all the atoms were relaxed. The SANDER (Simulated Annealing with NMR Derived Energy Restraints) module in the AMBER16 program was utilized to perform the four-step minimization before the MD simulation. In all minimization processes, 2000 steps for steepest descent method and 3000 steps for the conjugated gradient method were used, followed by application of the Particle Mesh Ewald Molecular Dynamics module in the MD simulation [33]. The system was heated from 10 to 300 K in 30 ps. The subsequent release process was similar to the minimization. Finally, all the atoms were relaxed at 300 K and 1 atm by applying periodic boundary conditions and equilibration for ~50 ps before the production run of 3.99 ns.

### 2.4. QSAR, ADME, and Toxicity Prediction

Quantitative structure-to-activity relationship (QSAR) properties were estimated using Molsoft and the SwissADME web servers [38]. Toxicity predictions were obtained from the ADMETlab web server [39]. For the best four performing ligands (cryptomisrine, cryptospirolepine, cryptoquindoline, and biscryptolepine), the parameters investigated included absorption, distribution, metabolism, elimination (ADME), and toxicity (T). ADME/T properties of lopinavir, omeprazole, and ibuprofen were computed for control purposes.

## 3. Results and Discussion

The search for therapeutic agents to combat the current COVID-19 pandemic is of utmost importance to all nations of the world. The absence of vaccines and potent antiviral agents against SARS-CoV-2 and effective treatment regimens amongst other factors has led to a significant increase in global mortality due to COVID-19. To date, the major strategy for identifying new COVID-19 drug candidates has been via drug repurposing, aided by high throughput screening of existing antiviral agents. Reliance on plant-derived natural products and formulations for the treatment of various diseases has proven beneficial globally. Extracts and compounds isolated from many of these plants have been shown to possess various pharmacological activities. *Cryptolepis sanguinolenta* is one such plant with antiviral, antiplasmodial, anti-inflammatory, and hypotensive capabilities [17]. Due to its antiviral potential where it has been suggested that it interferes with the viral replication machinery of herpes simplex virus [22], and the fact that this plant is present in many approved herbal preparations in Ghana, we sought to evaluate the potential of the alkaloids isolated from the plant as potential inhibitors of proteins expressed by SARS-CoV-2 in *in silico* studies.

### 3.1. Protein Targets

The protein targets selected were the main protease and the RNA-dependent RNA polymerase primarily due to the key roles these proteins play in viral replication and sustainability as well as the availability of experimental structures in the protein database. Two different groups have recently solved the structures of the main protease of SARS-CoV-2 [7, 8]. These 2 structures have been given the PDB codes 6LU7 and 6M0K. 6LU7 was chosen for this study because of its better resolution (1.5 Å) and the presence of a known *in vitro* inhibitor in the crystal structure. The presence of the ligand provides a simple route for the identification of binding pockets, selection of grid coordinates, and validation of docking protocols. The active site residues of this protease with a Cys-His catalytic dyad were identified. Two different structures were used for the RNA-dependent RNA polymerase. Initially, an experimental 3D structure of this protein was unavailable and was only obtained via homology modeling. A valid, high quality homology model of the RNA-dependent RNA polymerase was constructed by the SWISS-MODEL web server. The sequence similarity between RNA-dependent RNA polymerase and the template, 6NUR, on which the homology model was based was 96.35% [24, 25]. The validity of the model is evidenced by the fact that for the Ramachandran plot, 100% of the residues were in the allowed regions and 97.5% were in the most favored region. The utility of the homology model of the RNA-dependent RNA polymerase (RdRp) has been presented in a number of molecular docking literature [40, 41]. Recently, an electron microscopy structure of the RNA-dependent RNA polymerase (RdRpol) has been published [26]. Comparison of RdRpol to RdRp indicates high similarities within chain A (nsp12), and the root mean square deviation (RMSD) between 791 pruned atom pairs was 0.515 Å and that of 803 atom pairs was 0.933 Å (Fig. S1 A) indicating over 96% similarity and about 3% dissimilarity. Despite the similarities in the binding pockets of both RdRp and RdRpol, both structures were chosen for docking studies for comparison.

### 3.2. Validation of Docking Protocols

To ensure that the docking methods used in this study are suitable, a number of checks were employed. The SARS-CoV-2 main protease, M^pro^, was docked against N3. N3 is the ligand co-crystallized with M^pro^ in the crystal structure. Comparison of its docked output with the native revealed comparable pocket interactions. Interactions of N3 with Met49, Thr190, Gln189, Glu166, Ala191, Leu141, His41, Met165, His41, His163, and Gly143 were identified in both conformations (Fig. S2) covering significant catalytic subdomains in the binding pocket. Ten (10) conventional hydrogen bonds (distances between 2.3 and 3.7 Å) and eight (8) hydrophobic interactions were observed in the docking output. The hydrophobic interactions included amide pi-stacked, alkyl, and pi-alkyl interactions. Hydrogen bonding interactions existed predominantly between amino acids and amide moieties in N3 with active site residues of M^pro^. However, the orientation of the ligand was inverted in the docking output when compared to the crystal structure. A binding score of -7.3 kcal/mol was recorded which was consistent to that obtained by Huynh and coworkers [42] (Table 1).

Both RNA-dependent RNA polymerase structures used, RdRp and RdRpol, had similar binding pockets. Since no bound ligand was present in the RdRpol structure, ATP and a nucleotide analog, remdesivir, were chosen as ligands for validation purposes. The binding affinity of ATP towards RdRp was computed to be -7.4 kcal/mol whereas remdesivir towards RdRp was -6.9 kcal/mol. Both remdesivir (-7.3 kcal/mol) and ATP (-7.4 kcal/mol) had similar binding affinities towards RdRpol. Interactions observed in the docking of the two ligands with RdRp and RdRpol were also similar (Fig. S3).

Finally, docking of some ligands reported in the literature against the main protease or the RNA-dependent RNA polymerase was also carried out. The binding affinities obtained from the docking of these ligands with M^pro^ and RdRp and those reported were similar. Slight differences in results may be attributed to variation in software and computing power [43]. Ligand efficiencies were also found to be similar (Table 1). Hydroxychloroquine exhibited very low binding energies towards the main protease and the two RNA-dependent RNA polymerases used in this study (-6.2 kcal/mol for M^pro^, -5.4 kcal/mol for RdRp, and -5.9 kcal/mol for RdRpol), as shown in Table 1.

### 3.3. Docking of Alkaloids from *Cryptolepis sanguinolenta*

**Figure 2:**
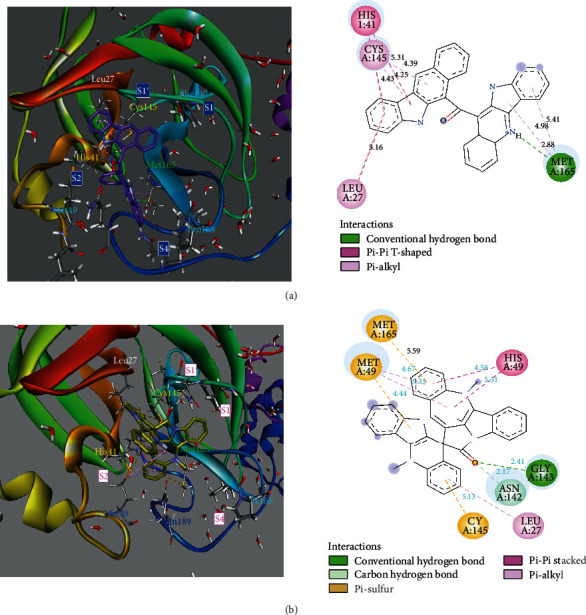
(a) View of 3D interaction of cryptomisirine with M^pro^ pocket residues (left with black labels) and 2D interactions colored by interaction type (right). (b) View of 3D interaction of cryptospirolepine with M^pro^ pocket residues (left with black labels) and 2D interactions colored by interaction type explained in legend (right).

Thirteen alkaloids isolated from *Cryptolepis sanguinolenta* were docked against M^pro^, RdRp, and RdRpol to identify possible binding interactions between proteins and the alkaloids. Against M^pro^, the binding energies observed for all 13 alkaloids were between -6.9 and -10.6 kcal/mol (Table 2). Cryptomisrine, cryptospirolepine, cryptoquindoline, and biscryptolepine possessed the best binding affinities, with binding energies less than -8.50 kcal/mol. In contrast, the binding energies of hydroxycryptolepine, cryptolepinone, neocryptolepine, isocryptolepine, quindoline, and cryptolepine were greater than -7.50 kcal/mol. The extent of association between the ligands and M^pro^ was estimated using the dissociation constant, *K*_*d*_. Cryptomisrine and cryptospirolepine had dissociation constants less than 100 nM, implying very strong association. Ligand efficiency, which represents the binding energy of the ligand per atom, was also calculated. Ligand efficiency of the ligands ranged between -0.26 and -0.41. All ligands interacted favorably and strongly between domains I and II of M^pro^ where the cysteine-histidine catalytic residues exist [44]. This pocket was observed to be hydrophobic with solvent accessible regions existing at the periphery. Of the best four ligands that interacted well with M^pro^, cryptomisrine and cryptospirolepine interacted with both Cys145 and His41. The nature of the interactions included pi-alkyl, pi-pi T-shaped pi-sulfur, and pi-pi stacked hydrophobic interactions with distances between 4 and 5 Å (Figure 2).

Hydrogen bonding was observed for the interaction between the quinoline nitrogen of cryptomisrine and Met165 of M^pro^ at a distance of 2.88 Å and at angles 116.352° and 102.64° (D-H-A and H-A-Y, respectively) leading to a resultant positively charged nitrogen (Figure 2). For cryptospirolepine, a conventional hydrogen bonding was found between the carbonyl oxygen and Gly143 at a distance of 2.41 Å (Figure 2) and angles 142.895° and 124.549° (DHA and HAY, respectively) whereas that with Asn142 was that of a carbon hydrogen bond at a distance of 2.17 Å and angles 131.604°and 138.885° (D-H-A and H-A-Y, respectively). Cryptoquindoline and biscryptolepine interacted with Met165 and Met49 via pi-alkyl hydrophobic forms at a distance between 4 and 5 Å whereas interactions with Glu166 were of a pi-sulfur and pi-anion kind. A conventional hydrogen bond was also found between Gln189 and biscryptolepine indole NH, at a distance of 1.91 Å (Fig. S4). Hydroxycryptolepine interacted strongly with the majority of the pocket residues showing very strong hydrogen bonding with Arg188 and Thr190 at distances between 2.13 and 2.8 Å (Fig. S4). All remaining ligands exhibited strong interactions with Cys145 and/or His41 as well as other residues in the various protease subdomains (Fig. S2).

When compared to other antiviral compounds suggested as possible lead compounds for COVID-19, the alkaloids of *Cryptolepis sanguinolenta* had noteworthy binding energies. For example, the binding energies of lopinavir, nelfinavir, and hydroxychloroquine when docked against M^pro^ were -8.7, -8.3, and -6.2 kcal/mol, respectively (Table 1). Low binding energies were associated with the binding of all the *Cryptolepis sanguinolenta* alkaloids against M^pro^. Of the 13 compounds tested, only cryptolepine had a binding energy greater than -7.0 kcal/mol (Table 2). The interaction of all alkaloids with at least one of the catalytic residues of M^pro^ suggests a probable mode of inhibition where these active site residues required for proteolysis are unavailable in the presence of the substrate. Recently, Gyebi and coworkers docked various alkaloids against the main protease of SARS-CoV-2, SARS-CoV, and MERS-CoV [45]. Interestingly, some alkaloids from *Cryptolepis sanguinolenta* exhibited strong binding to SARS-CoV-2 M^pro^, similar to the results obtained in this work. Estimated binding affinities were similar to that obtained in this work. Cryptospirolepine, with a binding affinity of -9.2 kcal/mol, was found to interact with Cys145. This is in agreement to the results of this work where cryptospirolepine interacted with both amino acids in the catalytic dyad—Cys145 and His41—with a binding affinity of -9.5 kcal/mol.

**Figure 3:**
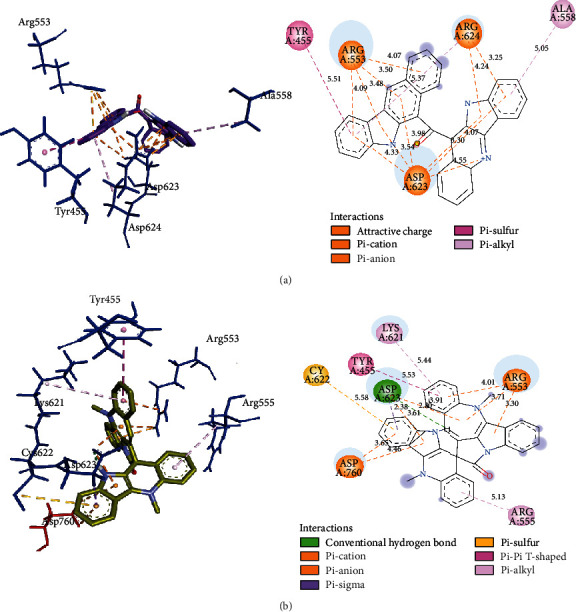
(a) View of 3D interaction of cryptomisrine with RdRp pocket residues (left with black labels) and 2D interactions colored by interaction type (right). (b) View of 3D interaction of cryptospirolepine with RdRp pocket residues (left with black labels) and 2D interactions colored by interaction type explained in legend (right).

**Figure 4:**
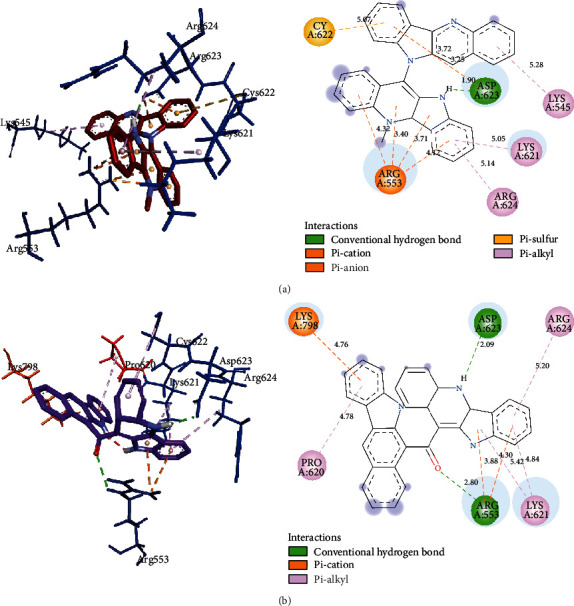
(a) View of 3D interaction of cryptoquindoline with RdRpol pocket residues (left with black labels) and 2D interactions colored by interaction type (right). (b) View of 3D interaction of cryptomisrine with RdRpol pocket residues (left with black labels) and 2D interactions colored by interaction type explained in legend (right).

In general, all the alkaloids bind to essential domains in the active sites of the two structures of the RNA-dependent RNA polymerase (Fig. S1 B and C). The differences in binding energy of alkaloids with both RdRp and RdRpol were about 0.5 kcal/mol. Of note, the binding energies of cryptolepicarboline, 11-isopropylcryptolepine, cryptoheptine, hydroxycryptolepine, cryptolepinone, neocryptolepine, and isocryptolepine with both RdRp and RdRpol were exactly the same (Table 2). Cryptomisrine, cryptospirolepine, cryptoquindoline, and biscryptolepine alkaloids exhibited strong binding to RdRp and RdRpol, with binding energies less than -8.50 kcal/mol. The binding constants (*K*_*d*_) for cryptomisrine, cryptospirolepine, cryptoquindoline, and biscryptolepine were well below 1 *μ*M for both RdRp and RdRpol, indicating strong association between ligands and protein targets. Cryptomisrine and cryptospirolepine exhibited much stronger binding to M^pro^ in contrast to both RdRp and RdRpol. Ligand efficiencies for all 13 alkaloids were very similar for M^pro^, RdRp, and RdRpol (Table 2). Electrostatic and hydrophobic interactions were the driving force of binding, in general. A positive charge on an aryl nitrogen of cryptomisrine interacted with the negative charge of an oxygen in Asp623 at a distance of 4.5 Å (Figure 3). The guanidino groups of Arg553 and Arg624 were involved in pi-cation interactions with pi-orbitals in cryptomisrine. Other common interactions observed were pi-anion, pi-pi T-shaped, and pi-alkyl interactions. Overall, 13 non-bonding interactions were obtained for cryptospirolepine and cryptoquindoline towards RdRp whereas 11 were found for biscryptolepine. Most of the interactions between alkaloids and RdRpol (Figure 4) were similar to those of RdRp, with residues in the palm, interface, and thumb domains (Fig. S1).

Elfiky recently described ribavirin, remdesivir, sofosbuvir, galidesivir, and tenofovir as potentially potent inhibitors of the RNA-dependent RNA polymerase of SARS-CoV-2 as they exhibited binding energies of -7.0 to -8.0 kcal/mol and suggested that the tight binding may inhibit polymerase function [40]. In a similar molecular docking study by Shah and coworkers, binding energies between -6.0 and -9.0 kcal/mol were obtained by docking a series of antiviral agents against different structures of the SARS-CoV-2 RNA-dependent RNA polymerase [41]. In comparison to both works, the alkaloids studied in this work showed much tighter binding as evidenced by the low binding energies. In particular, the high binding affinities of cryptomisrine, cryptospirolepine, cryptoquindoline, and biscryptolepine towards both RdRp and RdRpol makes them potential inhibitors that can be further explored.

### 3.4. Molecular Dynamics

**Figure 5:**
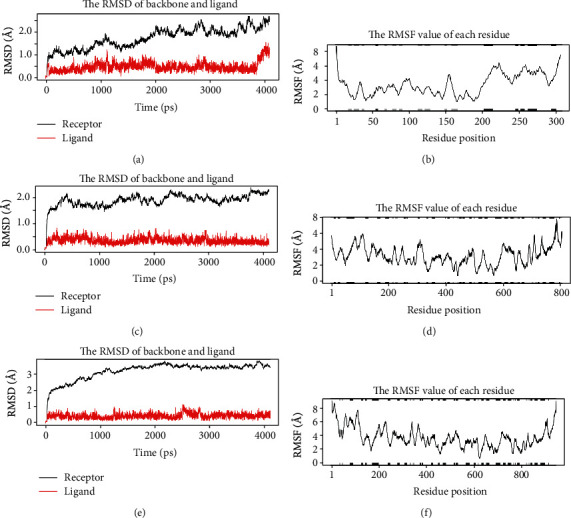
Room mean square deviation (RMSD) of cryptomisrine-M^pro^ (a), cryptomisrine-RdRp (c), and cryptomisrine-RdRpol (e) complexes. Root mean square fluctuation (RMSF) of cryptomisrine-M^pro^ (b), cryptomisrine-RdRp (d), and cryptomisrine-RdRpol (f) complexes. The secondary structure schematic added to the top and bottom margins of the figure shows helices as black, strands as gray, and loops as white, with larger fluctuations predicted for loop regions.

Ligand-driven molecular dynamics simulations were carried out to explore the stability of the interactions between ligands and proteins. The explicit water solvation model was applied to the four best ligands of M^pro^ and three best ligands of RdRp and RdRpol.The ligand binding free energies were estimated using both the Poisson-Boltzmann and the Generalized Born surface area continuum solvation, and the observations were summarized in Table 3 (detailed energetic contributions for binding free energy computations can be found in Table S1). For M^pro^, ligands investigated were cryptomisrine, cryptospirolepine, cryptoquindoline, and biscryptolepine. The root mean square deviation (RMSD) of ligands in the M^pro^-ligand complexes was between 0.39 and 0.62 Å and that of the protein (M^Pro^) was in the range of 1.43–1.95 Å. The radius of gyration for all M^pro^-ligand complexes was about 22 Å. For both RdRp and RdRpol, the ligands involved in the protein-ligand complexes investigated were cryptomisrine, cryptospirolepine, and cryptoquindoline. The ligand and protein RMSD for the RdRp complexes ranged between 0.30 and 0.69 Å and 1.80 and 1.87 Å, respectively. For the RdRpol complexes, the ligand and protein RMSD ranged between 0.42-0.51 Å and 2.94-3.96 Å, respectively. It is interesting to note that protein RMSD was much greater in RdRpol complexes (>2 Å) than in both RdRp and M^pro^ complexes (Table 3). The binding pocket of M^pro^ is characterized by tight structural packing and is probably responsible for observation of a largely stable protein during the entire simulation timescale. The presence of a metal cofactor in RdRp (which was absent in RdRpol) may also have contributed to its conformational stability shown in minimal overall configurational entropy. The radius of gyration for RdRp and RdRpol complexes was about 29 Å and 32 Å, respectively. However, the radius of gyration for the cryptospirolepine-RdRpol complex was much higher, at 38.65 Å. Entropic estimation for experimental structures were positive in M^pro^ (13–22 kcal/mol) and RdRpol (15 to 24 kcal/mol), except for those of RdRp (Table S1>). More tightly bound ligands are expected to have high entropic costs than loosely bound ligands [46]. All alkaloids were tightly bound to respective protein targets with overall high entropic costs as a result of high hydrophobic free energy contributions to total binding free energies (Δ*G*/PBSA and GBSA). Free energy contribution arising from electrostatic interactions with pocket residues was less in contrast to that of remdesivir triphosphate (RemTP) affecting the end point free energies (89.22 and 32.7 kcal/mol, Table S1). The nature and kinds of interactions that mediated protein ligand binding were largely similar to those obtained in the docking experiments (Table 3). Overall, the protein ligand complexes probed in the molecular dynamics simulations were stable with enhanced ligand binding efficiencies as well as observed minimal protein conformational fluctuations over the experimental timescale (Figure 5).

### 3.5. QSAR, ADME, and Toxicity

The QSAR and ADMET property and probability predictions were made to assess the key drug-like qualities of some of the alkaloids used. Cryptomisrine had the most hydrogen bond donors followed by biscryptolepine and hydroxycryptolepine. Cryptoheptine had the ability to accept the most hydrogen bonds followed by cryptoquindoline and quindoline. With a similar molecular landscape, all 13 alkaloids were largely hydrophobic, with most being moderately soluble to poorly soluble. Only hyroxycryptolepine was predicted to be completely soluble. All ligands have the ability to cross the blood-brain barrier with most having high gastrointestinal absorption indices (Table 4). In the QSAR analyses, all alkaloids investigated violated either one or none of Lipinski's rules and hence were drug-like. Lopinavir, omeprazole, and ibuprofen were used for comparison in ADMET probabilities and predictions (Table 4 and Tables S2 and S3)). Despite the low aqueous solubility of the four alkaloids studied, their permeability as well as metabolism was relatively high compared to that of lopinavir. The volume distribution of the four (0.291 L/kg, 0.59 L/kg, 0.78 L/kg, and 0.625 L/kg) indicates a better distribution. The predictions show low half-life times indicating good bioavailability with relatively higher clearance rates (see Table S1). Drug-likeness model score predicted values between -1 and -0.2, on a scale of -6 (largely for non-drugs) to +6 (highest score for drugs) [47]. The majority, however, had scores from -0.20 to -0.5, indicating that these compounds are fairly drug-like [47, 48] confirming Lipinski's predictions. LD_50_'s greater than 500 mg/kg with the exception of cryptospirolepine (LD_50_; 184.902 mg/kg) were obtained for the best four tight binding alkaloids assessed. These three alkaloids were less toxic than lopinavir (LD_50_; 570.85 mg/kg). Overall, these predictions indicate that these molecules taken together would be safe. Cryptoquindoline was predicted to be strongly mutagenic (probability 0.8), cryptomisrine was moderate (probability, 0.6) while in contrast, cryptospirolepine and biscryptolepine were not potential mutagens or carcinogens (Table S2). Toxicity studies of the extracts and alkaloids of *Cryptolepis sanguinolenta* are well documented in the literature [49–52], and have led to investigations into their use as potential anticancer agents [52, 53]. It has been shown that the plant extract when administered at very high concentrations of 2000 mg/kg may result in some degree of toxicity but is safe at concentrations at or below 500 mg/kg [17, 51]. Investigations into tolerable dosages in animal models could be used to confirm our findings on toxicity, mutagenicity, and carcinogenicity.

## 4. Conclusion

The use of natural plant-based extracts as antiviral agents is well established in the literature. One advantage of using plant extracts for therapeutic purposes is the potential for synergism by the compounds of that extract. As shown in this molecular docking study, the different alkaloids of *Cryptolepis sanguinolenta* have exhibited high binding affinity and hence potential inhibitory activity towards two of the major prions of SARS-CoV-2, the main protease and the RNA-dependent RNA polymerase. The high binding affinity of cryptomisrine, cryptospirolepine, cryptoquindoline, and biscryptolepine for both protein targets, drug-like characteristics, tight binding, and residence times within protein binding pockets makes them ideal candidates for further *in vitro* and *in vivo* validation.

## Figures and Tables

**Table 1:** Comparison of binding free energies (Δ*G*) and ligand efficiencies of compounds in the literature and this study for validation purposes.

Ligand	Free energy of binding, Δ*G* (kcal/mol)					Ligand efficiency (Δ*g*)				
	Mpro	Mpro^∗^	RdRp	RdRp	RdRpol	Mpro	Mpro^∗^	RdRp	RdRp^∗^	RdRpol
N3	-7.3^#^	-8.37^1^	—	—	—	-0.15	-0.17	—	—	—
Nelfinavir	-8.3	-10.72^1^	—	—		-0.21	-0.27			
Luteolin-7-glucoside	-8.1	-8.17^1^	—	—	—	-0.25	-0.39	—	—	—
Curcumin	-7.0	-7.05^1^			—	-0.26	-0.26		—	—
ATP	—	—	-7.4	-7.2^2^	-7.4	—	—	-0.27	—	-0.27
Remdesivir	—	—	-6.9	-6.4^2^	-7.3	—	—	-0.17	—	-0.17
Lopinavir	-8.7	-9.41^1^	-7.8	—	-8.3	-0.19	-0.20	-0.17	—	-0.18
Hydroxychloroquine	-6.2	—	-5.4	—	-5.9	-0.27	—	-0.24	—	-0.26

^∗^Data extracted from literature; Δ*G* of native ligand using DINC 2.0; ^1^Data values reported from Reference [44]; ^2^Data values reported from Reference [54].

**Table 2:** Binding free energies (Δ*G*), binding constant (*K*_*d*_) and ligand efficiencies of *Cryptolepis sanguinolenta* alkaloids against SARS-CoV-2 viral proteins.

Ligand	Free energy of binding, Δ*G* (kcal/mol)			Binding constant, *K*_*d*_ (*μ*M)			Ligand efficiency (Δ*g*)		
	Mpro	RdRp	RdRpol	Mpro	RdRp	RdRpol	Mpro	RdRp	RdRpol
Cryptomisrine	-10.60	-9.80	-9.40	0.033	0.120	0.238	-0.29	-0.27	-0.26
Cryptospirolepine	-10.00	-9.10	-9.20	0.0897	0.386	0.329	-0.27	-0.25	-0.25
Cryptoquindoline	-9.50	-8.75	-9.70	0.202	0.682	0.146	-0.29	-0.26	-0.29
Biscryptolepine	-8.80	-8.90	-9.10	0.628	0.535	0.386	-0.24	-0.25	-0.25
Cryptolepicarboline	-8.20	-8.45	-8.45	1.665	1.110	1.110	-0.26	-0.27	-0.27
11-Isopropylcryptolepine	-7.80	-7.30	-7.30	3.186	7.171	7.171	-0.37	-0.35	-0.35
Cryptoheptine	-7.80	-7.20	-7.20	3.186	8.434	8.434	-0.41	-0.38	-0.38
Hydroxycryptolepine	-7.20	-6.80	-6.80	8.434	16.141	16.141	-0.38	-0.36	-0.36
Cryptolepinone	-7.20	-7.05	-7.05	8.434	10.758	10.758	-0.38	-0.37	-0.37
Neocryptolepine	-7.20	-7.00	-7.00	8.434	11.668	11.668	-0.40	-0.39	-0.39
Isocryptolepine	-7.10	-7.00	-7.00	9.92	11.668	11.668	-0.39	-0.39	-0.39
Quindoline	-7.00	-7.60	-7.10	11.668	4.407	9.920	-0.41	-0.45	-0.41
Cryptolepine	-6.90	-7.05	-6.70	13.723	10.758	18.984	-0.38	-0.39	-0.37

M^pro^: main protease of SARS-CoV-2; RdRp: homology model of RNA-dependent RNA polymerase of SARS-CoV-2; RdRpol: electron microscopy model of RNA-dependent RNA polymerase of SARS-CoV-2.

**Table 3:** Ligand-driven molecular dynamics simulation data of ligands with best binding affinities recorded from docking study using explicit water model.

P-L complex	RMSD (avg.) (Å)		Rg (Å)	*Δ*PBSA_bind_ (kcal/mol)	*Δ*GBSA_bind_ (kcal/mol)	H-bonding^∗^	Dist. (Å)	Ang. (°)
	Ligand	Protein						
M^pro^								
Cryptospirolepine	0.39	1.95	22.17	-15.65	-22.87	Gly^143^, OH	3.04	154.02
Cryptomisirine	0.51	1.74	22.19	-14.32	-24.37	Arg^188^, N-H	3.30	143.14
Biscryptolepine	0.60	1.43	22.14	-12.68	-21.16	Gln^189^, N-H	3.29	147.33
Cryptoquindoline	0.62	1.67	22.03	-8.43	-15.14	Gln^189^, N-H	3.27	153.77
RdRp								
Cryptomisirine	0.30	1.87	28.59	-53.54	-60.15	Thr^556^, N-H	3.06	152.95
Cryptospirolepine	0.68	1.85	28.76	-44.94	-54.45	Asn^691^, N	3.20	141.31
Cryptoquindoline	0.69	1.80	28.63	-44.91	-55.68	Lys^621^, N-H	3.33	151.55
RemTP	0.47	1.94	28.78	89.22	32.7	Asp^623^, O-H	3.07	149.90
RdRpol								
Cryptospirolepine	0.51	2.94	32.37	-17.07	-20.35	Arg^553^, N-H	3.27	143.58
Cryptomisirine	0.42	3.16	32.05	-12.24	-16.92	Asp^623^, N-H	3.13	151.01
Cryptoquindoline	0.47	2.99	38.65	-4.98	-10.66	Lys^621^, N-H	3.35	148.11

RMSD (avg): average root mean square deviation; Rg: average radius of gyration; *Δ*PBSA: binding free energy using Poisson-Boltzmann surface area continuum solvation; *Δ*GBSA: binding free energy Generalized Born surface area continuum solvation; H-bonding: most frequent interacting pocket residue; Dist.: average distance; Ang.: average interaction angle.

**Table 4:** QSAR and drug-likeness profile predicted for the *Cryptolepis sanguinolenta* alkaloids from the SwissADME and ADMETlab web servers.

Ligand	MW	HBA	HBD	TPSA	cLogP_o/w_	ESOL logs	ESOL class	GI abs.	LogK_p_ (S.P.)	LP.V	LD.V	SA	*D*-score
11-Isopropylcryptolepine	276.38	0	1	19.03	4.11	-5.1	Moderately soluble	High	-4.51	0	1	3.5	-0.62
Biscryptolepine	468.59	0	2	38.06	5.23	-7.37	Poorly soluble	High	-4.42	1	2	5.46	-0.86
Cryptoheptine	246.26	3	1	46.01	2.95	-4.06	Moderately soluble	High	-5.56	0	1	2.43	-0.49
Cryptolepicarboline	397.47	1	0	22.75	5.57	-6.99	Poorly soluble	Low	-4.18	1	2	2.99	-0.99
Cryptolepine	232.28	1	0	17.82	3.29	-4.08	Moderately soluble	High	-5.35	0	1	1.71	-0.69
Cryptolepinone	248.28	1	1	37.79	2.97	-4.29	Moderately soluble	High	-5.28	0	2	2.08	-0.20
Cryptomisrine	468.57	1	3	56.92	4.94	-8.00	Poorly soluble	High	-3.70	1	2	5.01	-0.48
Cryptoquindoline	448.52	2	0	35.64	5.92	-7.53	Poorly soluble	Low	-4.24	1	2	3.2	-1.18
Cryptospirolepine	504.58	1	1	45.96	5.63	-7.64	Poorly soluble	Low	-4.82	0	2	4.95	-0.34
Hydroxycryptolepine	250.3	1	2	39.26	2.47	-3.65	Soluble	High	-5.95	0	0	3.17	-0.24
Isocryptolepine	232.28	1	0	17.82	3.25	-4.05	Moderately soluble	High	-5.38	0	1	1.44	-0.84
Neocryptolepine	232.28	1	0	17.82	3.47	-4.32	Moderately soluble	High	-5.08	0	2	1.56	-0.38
Quindoline	217.25	2	0	25.78	1.94	-4.17	Moderately soluble	High	-5.10	0	2	1.57	-1.03

MW: molecular weight; HBA: hydrogen bond acceptor; HBD: hydrogen bond donor; TPSA: topological polar surface area; cLogP_o/w_: lipophilicity; ESOL logs: water solubility; ESOL class: classification of water solubility; GI abs.: gastrointestinal absorption; LogK_p_ (S.P.): skin permeability; LP.V: number of Lipinski's rules violated; LD.V: lead-likeness violation; SA: synthetic ability; *D*-score: drug-likeness model score. All alkaloids violated none or only one of Lipinski's rules. Cryptomisirine donates the most hydrogen bonds followed by biscryptolepine and hydroxycryptolepine. Cryptoheptine had the ability to accept the most hydrogen bonds followed by cryptoquindoline and quindoline. With a similar molecular landscape, all 13 alkaloids were largely hydrophobic, with most being moderately soluble to poorly soluble. Only hydroxycryptolepine was completely soluble. All ligands have the ability to cross the blood-brain barrier with most having high gastrointestinal absorption indices.

## Data Availability

All data generated or analyzed during this study are available upon reasonable request.

## Supplementary Materials

Supplementary Materials 2D and 3D interactions of some protein-ligand complexes are available as supplementary materials. ADMET predictions for ligands and some drugs are also included.

## References

[1] WHO (2020). WHO Director-General’s opening remarks at the media briefing on COVID-19 - 11 March 2020. https://www.who.int/dg/speeches/detail/who-director-general-s-opening-remarks-at-the-media-briefing-on-covid-19---11-march-2020.

[2] Coronaviridae Study Group of the International Committee on Taxonomy of Viruses (2020). The species severe acute respiratory syndrome-related coronavirus: classifying 2019-NCoV and naming it SARS-CoV-2. *Nature Microbiology*.

[3] Wu F., Zhao S., Yu B. (2020). A new coronavirus associated with human respiratory disease in China. *Nature*.

[4] Monto A. S., Cowling B. J., Peiris J. S. M. (2014). Coronaviruses. *In Viral Infectios of Humans*.

[5] Shereen M. A., Khan S., Kazmi A., Bashir N., Siddique R. (2020). COVID-19 infection: origin, transmission, and characteristics of human coronaviruses. *Journal of Advanced Research*.

[6] Masters P. S. (2006). The molecular biology of coronaviruses. *Advances in Virus Research*.

[7] Dai W., Zhang B., Jiang X. M. (2020). Structure-based design of antiviral drug candidates targeting the SARS-CoV-2 main protease. *Science*.

[8] Jin Z., Du X., Xu Y. (2020). Structure of M^pro^ from SARS-CoV-2 and discovery of its inhibitors. *Nature*.

[9] Xue X., Yu H., Yang H. (2008). Structures of two coronavirus main proteases: implications for substrate binding and antiviral drug design. *Journal of Virology*.

[10] Pillaiyar T., Manickam M., Namasivayam V., Hayashi Y., Jung S.-H. (2016). An overview of severe acute respiratory syndrome–coronavirus (SARS-CoV) 3CL protease inhibitors: peptidomimetics and small molecule chemotherapy. *Journal of Medicinal Chemistry*.

[11] Lehmann K. C., Gulyaeva A., Zevenhoven-Dobbe J. C. (2015). Discovery of an essential nucleotidylating activity associated with a newly delineated conserved domain in the RNA polymerase-containing protein of all nidoviruses. *Nucleic Acids Research*.

[12] Yin W., Mao C., Luan X. (2020). Structural basis for inhibition of the RNA-dependent RNA polymerase from SARS-CoV-2 by remdesivir. *Science*.

[13] Park M., Thwaites R. S., Openshaw P. J. M. (2020). COVID‐19: lessons from SARS and MERS. *European Journal of Immunology*.

[14] WHO (2005). *National policy on traditional medicine and regulation of herbal medicines: report of a WHO global survey*.

[15] Bierer D. E., Dubenko L. G., Zhang P. (1998). Antihyperglycemic activities of cryptolepine analogues: an ethnobotanical lead structure isolated from Cryptolepis sanguinolenta†. *Journal of Medicinal Chemistry*.

[16] Cimanga K., De Bruyne T., Pieters L., Vlietinck A. J., Turger C. A. (1997). In vitro and in vivo antiplasmodial activity of cryptolepine and related alkaloids from Cryptolepis sanguinolenta. *Journal of Natural Products*.

[17] Osafo N., Mensah K. B., Yeboah O. K. (2017). Phytochemical and pharmacological review of *Cryptolepis sanguinolenta* (Lindl.) Schlechter. *Advances in Pharmacological Sciences*.

[18] Paulo A., Gomes E. T., Steele J., Warhurst D. C., Houghton P. J. (2000). Antiplasmodial activity of Cryptolepis sanguinolenta alkaloids from leaves and roots. *Planta Medica*.

[19] Paulo A., Pimentel M., Viegas S. (1994). Cryptolepis sanguinolenta activity against diarrhoeal bacteria. *Journal of Ethnopharmacology*.

[20] Boakye-Yiadom K. (2008). Antimicrobial properties of some west African medicinal plants II. Antimicrobial activity of aqueous extracts ofCryptolepis Sanguinolenta(Lindl.) Schlechter. *Quarterly Journal of Crude Drug Research*.

[21] Tona L., Kambu K., Ngimbi N., Cimanga K., Vlietinck A. J. (1998). Antiamoebic and phytochemical screening of some Congolese medicinal plants. *Journal of Ethnopharmacology*.

[22] Silva O., Barbosa S., Diniz A., Valdeira M. L., Gomes E. (1997). Plant extracts antiviral activity against herpes simplex virus type 1 and African swine fever virus. *International Journal of Pharmacognosy*.

[23] Laskowski R. A. (2001). PDBsum: summaries and analyses of PDB structures. *Nucleic Acids Research*.

[24] Kirchdoerfer R. N., Ward A. B. (2019). Structure of the SARS-CoV Nsp12 polymerase bound to Nsp7 and Nsp8 co-factors. *Nature Communications*.

[25] Waterhouse A., Bertoni M., Bienert S. (2018). SWISS-MODEL: homology modelling of protein structures and complexes. *Nucleic Acids Research*.

[26] Gao Y., Yan L., Huang Y. (2020). Structure of the RNA-dependent RNA polymerase from COVID-19 virus. *Science*.

[27] Webb B., Sali A. (2016). Comparative protein structure modeling using MODELLER. *Current Protocols in Bioinformatics*.

[28] Maier J. A., Martinez C., Kasavajhala K., Wickstrom L., Hauser K. E., Simmerling C. (2015). ff14SB: improving the accuracy of protein side chain and backbone parameters from ff99SB. *Journal of Chemical Theory and Computation*.

[29] Wang J., Wang W., Kollman P. A., Case D. A. (2006). Automatic atom type and bond type perception in molecular mechanical calculations. *Journal of Molecular Graphics and Modelling*.

[30] Antunes D. A., Moll M., Devaurs D., Jackson K. R., Lizée G., Kavraki L. E. (2017). DINC 2.0: a new protein–peptide docking webserver using an incremental approach. *Cancer Research*.

[31] Hopkins A. L., Groom C. R., Alex A. (2004). Ligand efficiency: a useful metric for lead selection. *Drug Discovery Today*.

[32] Yang J.-F., Wang F., Chen Y.-Z., Hao G.-F., Yang G.-F. (2019). LARMD: integration of bioinformatic resources to profile ligand-driven protein dynamics with a case on the activation of estrogen receptor. *Briefings in Bioinformatics*.

[33] Case D. A., Cheatham T. E., Darden T. (2005). The Amber biomolecular simulation programs. *Journal of Computational Chemistry*.

[34] Jakalian A., Jack D. B., Bayly C. I. (2002). Fast, efficient generation of high-quality atomic charges. AM1-BCC model: II. Parameterization and validation. *Journal of Computational Chemistry*.

[35] Wang J., Wolf R. M., Caldwell J. W., Kollman P. A., Case D. A. (2004). Development and testing of a general Amber force field. *Journal of Computational Chemistry*.

[36] Price D. J., Brooks C. L. (2004). A modified TIP3P water potential for simulation with Ewald summation. *The Journal of Chemical Physics*.

[37] Jorgensen W. L., Chandrasekhar J., Madura J. D., Impey R. W., Klein M. L. (1983). Comparison of simple potential functions for simulating liquid water. *The Journal of Chemical Physics*.

[38] Daina A., Michielin O., Zoete V. (2017). SwissADME: a free web tool to evaluate pharmacokinetics, drug-likeness and medicinal chemistry friendliness of small molecules. *Scientific Reports*.

[39] Dong J., Wang N.-N., Yao Z.-J. (2018). ADMETlab: a platform for systematic ADMET evaluation based on a comprehensively collected ADMET database. *Journal of Cheminformatics*.

[40] Elfiky A. A. (2020). Ribavirin, remdesivir, sofosbuvir, galidesivir, and tenofovir against SARS-CoV-2 RNA dependent RNA polymerase (RdRp): a molecular docking study. *Life Sciences*.

[41] Shah B., Modi P., Sagar S. R. (2020). In silico studies on therapeutic agents for COVID-19: drug repurposing approach. *Life Sciences*.

[42] Huynh T., Wang H., Luan B. (2020). In SilicoExploration of the Molecular Mechanism of Clinically Oriented Drugs for Possibly Inhibiting SARS-CoV-2’s Main Protease. *The Journal of Physical Chemistry Letters*.

[43] Pantsar T., Poso A. (2018). Binding affinity via docking: fact and fiction. *Molecules*.

[44] Khaerunnisa S., Kurniawan H., Awaluddin R., Suhartati S., Soetjipto S. (2020). Potential inhibitor of COVID-19 main protease (M^pro^) from several medicinal plant compounds by molecular docking study. *Preprints*.

[45] Gyebi G. A., Ogunro O. B., Adegunloye A. P., Ogunyemi O. M., Afolabi S. O. (2020). Potential inhibitors of coronavirus 3-chymotrypsin-like protease (3CLpro): anin silicoscreening of alkaloids and terpenoids from African medicinal plants. *Journal of Biomolecular Structure and Dynamics*.

[46] Swanson J. M. J., Henchman R. H., McCammon J. A. (2004). Revisiting free energy calculations: a theoretical connection to MM/PBSA and direct calculation of the association free energy. *Biophysical Journal*.

[47] Chen G., Zheng S., Luo X. (2005). Focused combinatorial library design based on structural diversity, druglikeness and binding affinity score. *Journal of Combinatorial Chemistry*.

[48] Karageorgis G., Foley D. J., Laraia L., Waldmann H. (2020). Principle and design of pseudo-natural products. *Nature Chemistry*.

[49] Ansah C., Mfoafo E. A., Woode E., Opoku-Okrah C., Owiredu W. K. B. A. (2008). Toxicological evaluation of the anti-malarial herb Cryptolepis sanguinolenta in rodents. *Journal of Pharmacology and Toxicology*.

[50] Ansah C., Otsyina H. R., Duwiejua M., Woode E., Aboagye F. A., Aning K. G. (2009). Toxicological assessment of Cryptolepis sanguinolenta for possible use in veterinary medicine. *Journal of Veterinary Medicine and Animal Health*.

[51] Ajayi A. F., Akhigbe R. E., Iyiola T. O., Adewumi O. M., Olaleye S. B. (2012). Gastric secretagogue action of Cryptolepis sanguinolenta in the perfused stomach of anesthetized rats. *International Journal of Medicine and Biomedical Research*.

[52] Ansah C., Gooderham N. J. (2002). The popular herbal antimalarial, extract of Cryptolepis sanguinolenta, is potently cytotoxic. *Toxicological Sciences*.

[53] Ansha C., Mensah K. B. (2013). A review of the anticancer potential of the antimalarial herbal *Cryptolepis sanguinolenta* and its major alkaloid cryptolepine. *Ghana Medical Journal*.

[54] Zhang L., Zhou R. (2020). Binding mechanism of remdesivir to SARS-CoV-2 RNA dependent RNA polymerase. *Preprints*.

