# Supplementary material for: Alkaloids from Cryptolepis sanguinolenta as Potential Inhibitors of SARS-CoV-2 Viral Proteins: An In Silico Study
Source: Biomed Res Int. 2020 Sep 22;2020:5324560. doi: 10.1155/2020/5324560 (PMC7512045; doi:10.1155/2020/5324560)
Supplement: Supplementary Materials — 2D and 3D interactions of some protein-ligand complexes are available as supplementary materials. ADMET predictions for ligands and some drugs are also included. [file 5324560.f1.docx]

**Alkaloids from *Cryptolepis sanguinolenta* as Potential Inhibitors of SARS – CoV – 2 Viral Proteins: an *in silico* study**

Lawrence Sheringham Borquaye^1,2*^, Edward Ntim Gasu^1,2^, Gilbert Ampomah Boadu^1^, Lois Kwane Kyei^2^, Margaret Amerley Amarh^1^, Caleb Nketia Mensah^1^, Daniel Nartey^1^, Michael Commodore^1^, Abigail Kusiwaa Adomako^1^, Philipina Acheampong^1^, Jehoshaphat Oppong Mensah^1^, David Batsa Mormor^1^ and Caleb Impraim Aboagye^1^

^1^Department of Chemistry, Kwame Nkrumah University of Science and Technology, Kumasi - Ghana

^2^Central Laboratory, Kwame Nkrumah University of Science and Technology, Kumasi – Ghana

Lawrence Sheringham Borquaye – lsborquaye.sci@knust.edu.gh, slborquaye@gmail.com

Edward Ntim Gasu – engasu24@gmail.com

Gilbert Ampomah Boadu – gilbertboadu@outlook.com

Lois Kwane Kyei – loiskyei.k@gmail.com

Margaret Amerley Amarh – margaret_amarh@yahoo.com

Caleb Nketia Mensah – caliptonketia@gmail.com

Daniel Nartey – danielnartey.nd12@gmail.com

Michael Commodore – michaelcommodore7@gmail.com

Abigail Kusiwaa Adomako – aadomako80@gmail.com

Philipina Acheampong – philipinacheampong32@gmail.com

Jehoshaphat Oppong Mensah – jehoshaphatmensah@gmail.com

David Batsa Mormor – mormordavid0@gmail.com

Caleb Impraim Aboagye – calebelocarm83@gmail.com

***Corresponding Author**

Lawrence Sheringham Borquaye

Department of Chemistry

Kwame Nkrumah University of Science and Technology

Kumasi – Ghana

Phone: +233-246-551-450

Email: lsborquaye.sci@knust.edu.gh, [slborquaye@gmail.com](mailto:slborquaye@gmail.com)


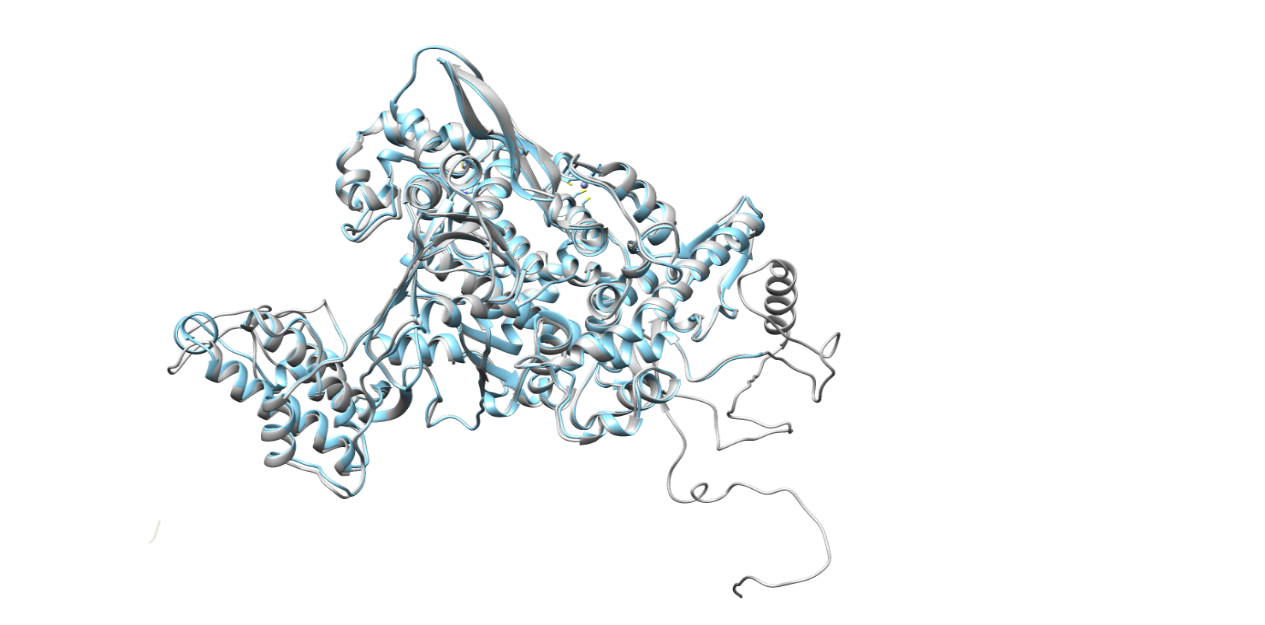


**A**


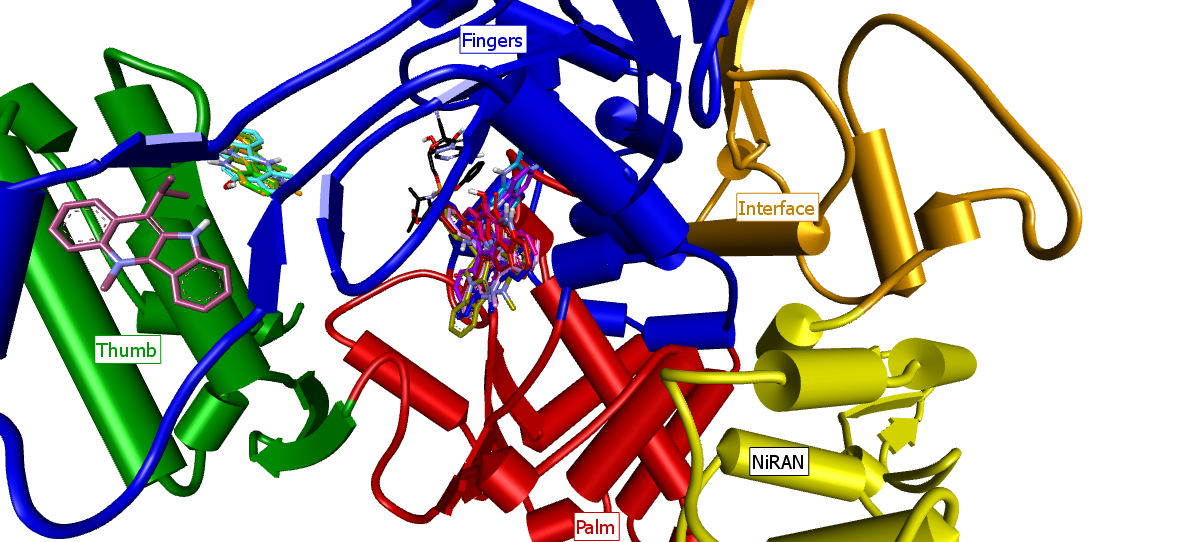


**B**


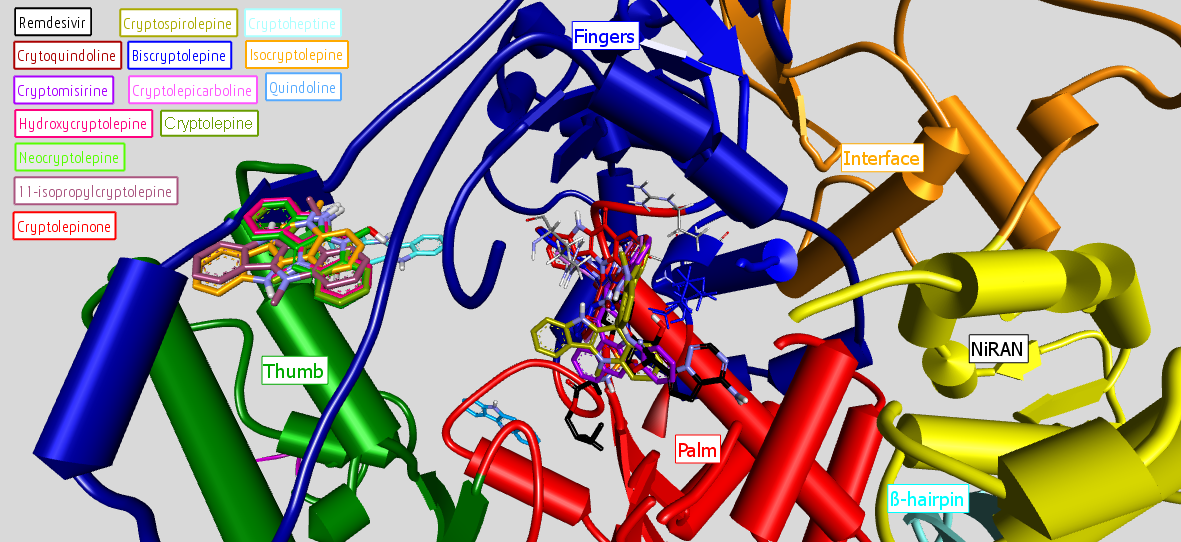


**C**

**Fig. S1**: (A) Structural matching for RNA – dependent RNA polymerase experimental and refined models. Dark gray is the experimental model which was refined and the light blue is the homology model. Arrows (black) show regions of deviation (B) remdesivir and all 13 alkaloids in the RdRp binding pocket and (C) remdesivir and most alkaloids in the palm and finger domains and a few others in the thumb domain of RdRpol.


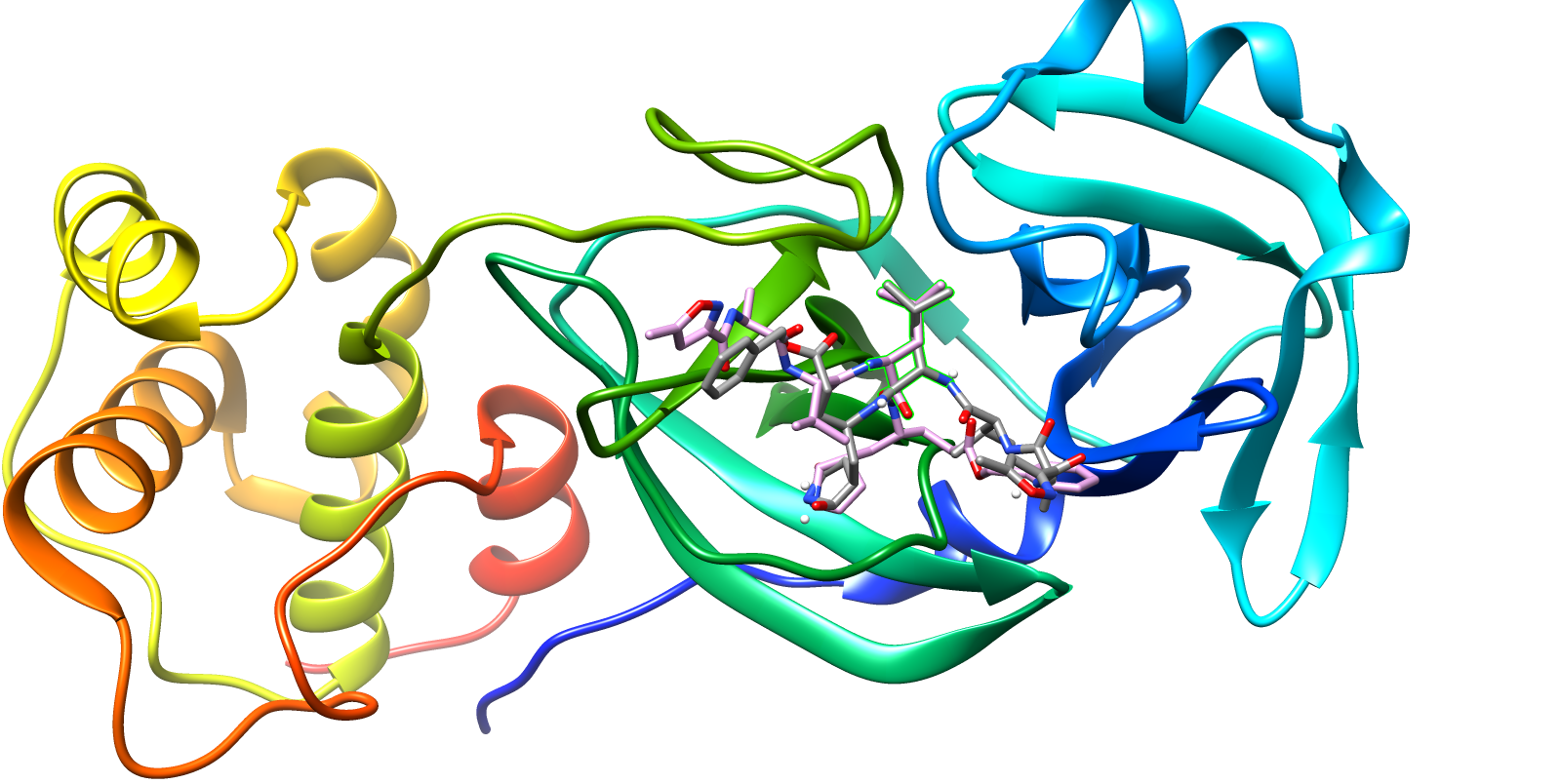

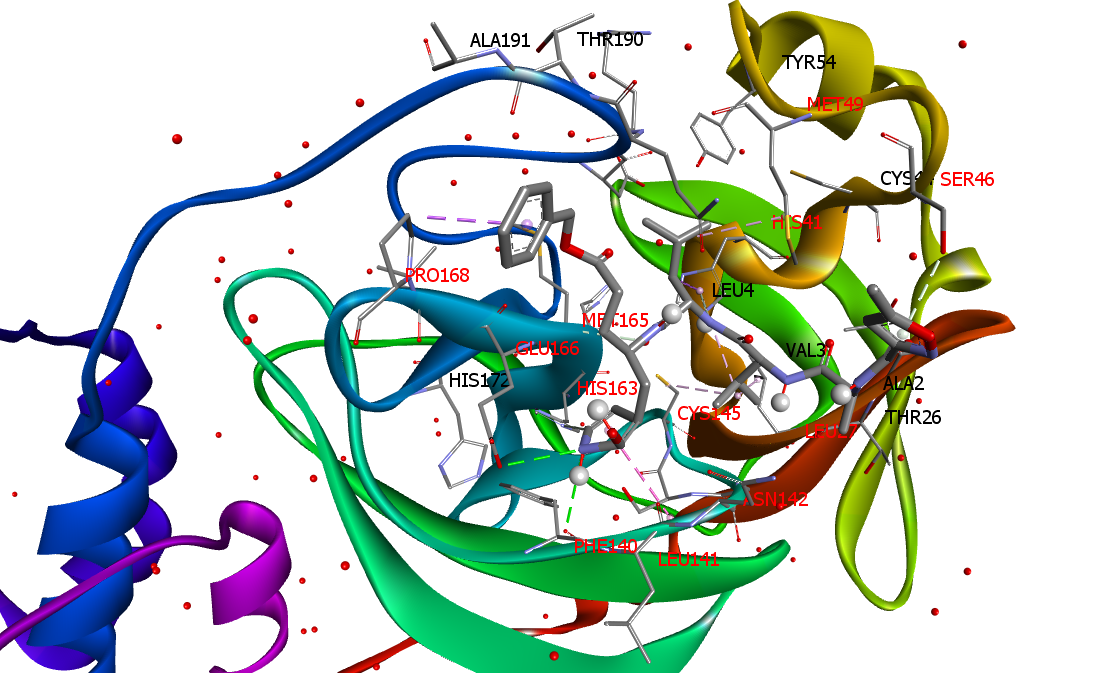

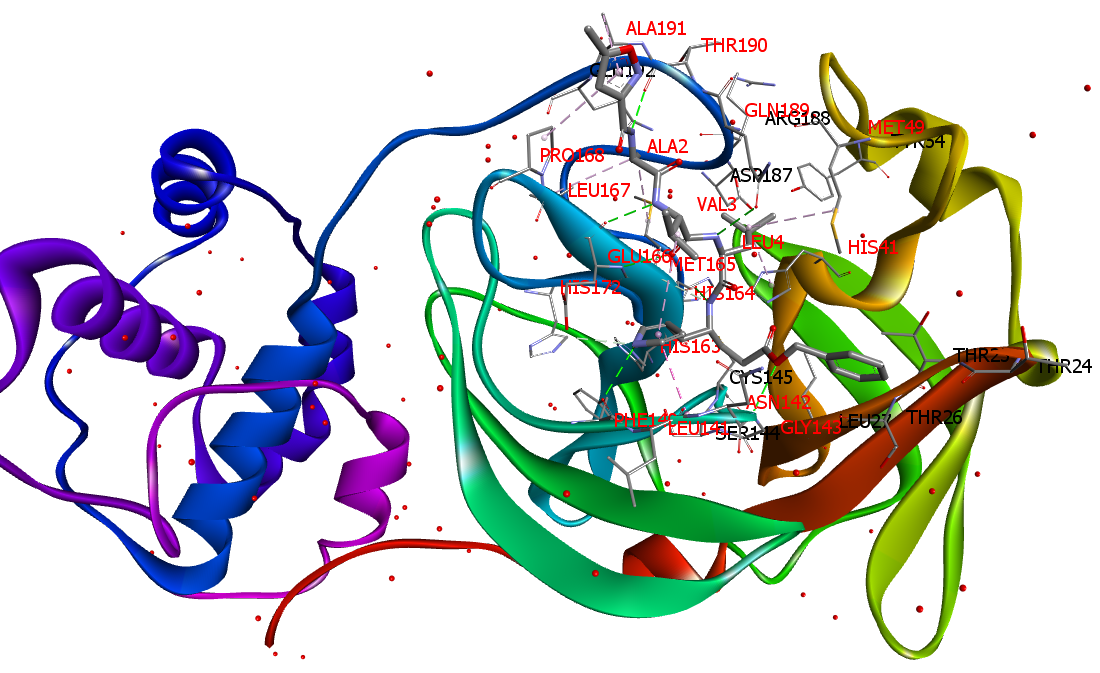


**A**

**B**

**C**


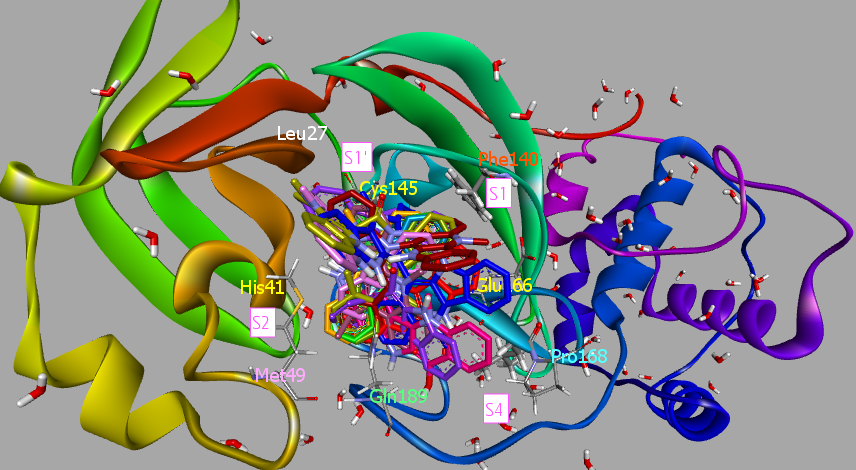


**D**

**Fig. S2:**

(**A**) Interactions of N3 with residues of M^pro^ in the crystal structure - A total of 18 favorable non-bonding interactions observed with 16 pocket residues for M^pro^ native N3

**(B**) N3 docked to M^pro^ - 13 favorable interactions were observed with 12 pocket residues

(**C**) Comparison of native N3 and docked conformations (pink and ash carbon backbone respectively), in M^pro^ binding site. Similar conformations were observed however, orientations inverted. Consensus region (green outline) gave RMSD of 1.452 Å.

(**D**) ribbon representation of M^pro^ showing catalytic sub-domains, S1, S1’, S2, and S3 and ligands in their best binding conformations.


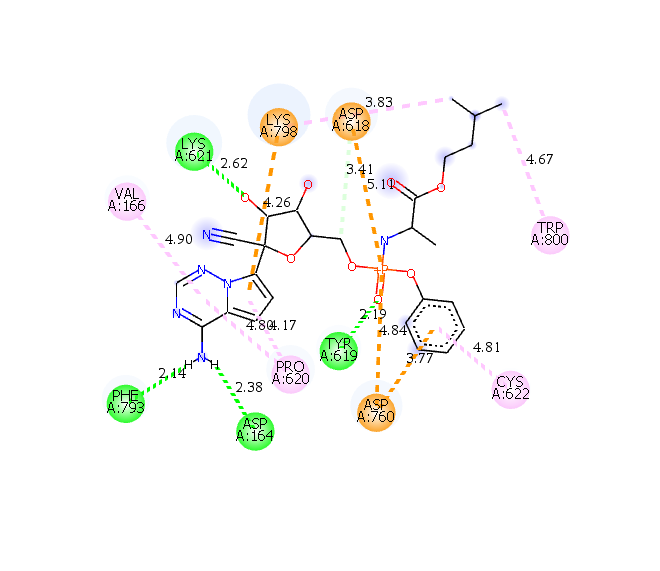

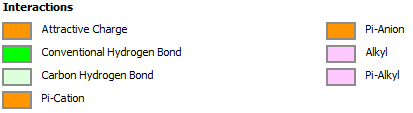

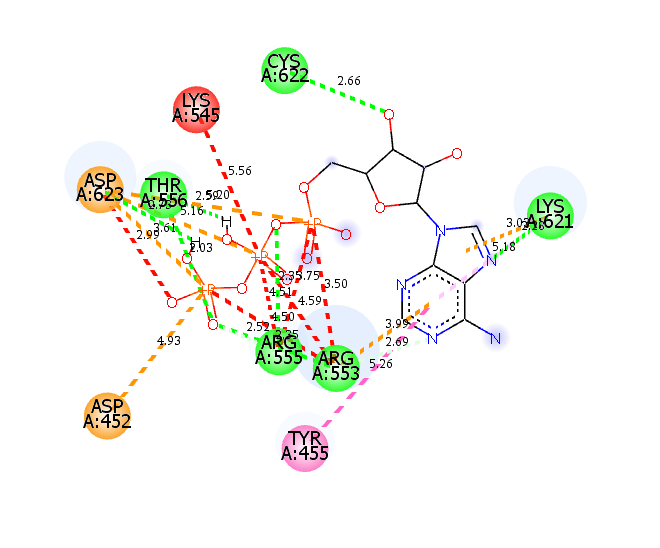

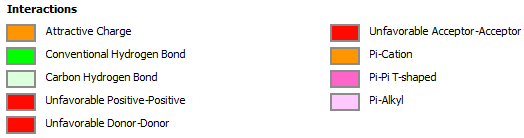


**A**

**B**

**Fig. S3:** Comparing the interactions of (A) remdesivir and (B) adenosine tri-phosphate. There was a similar number of favorable interactions, however remdesivir made extra contacts. Conventional hydrogen bonding interaction was only similar for Lys621.


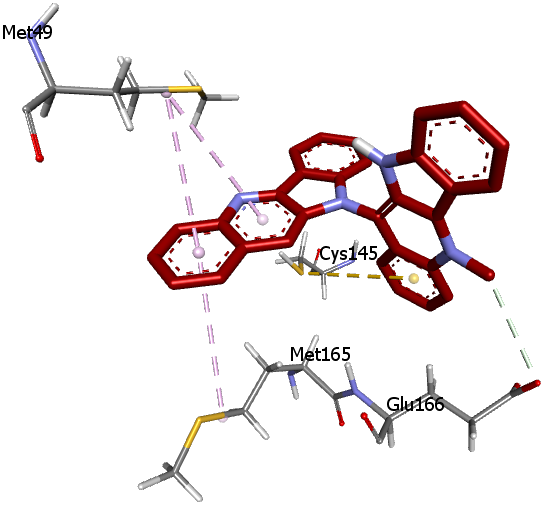


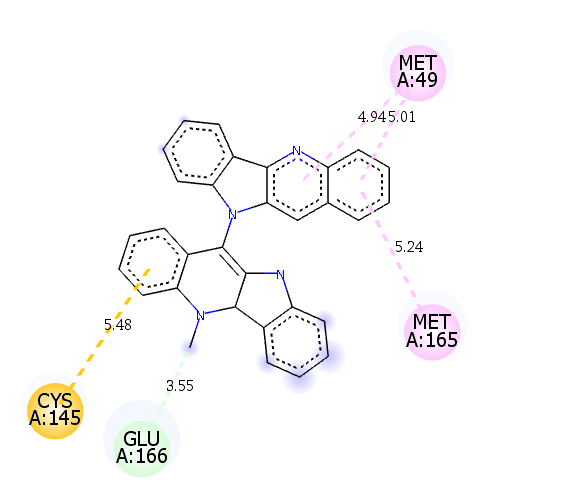

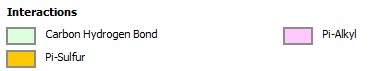

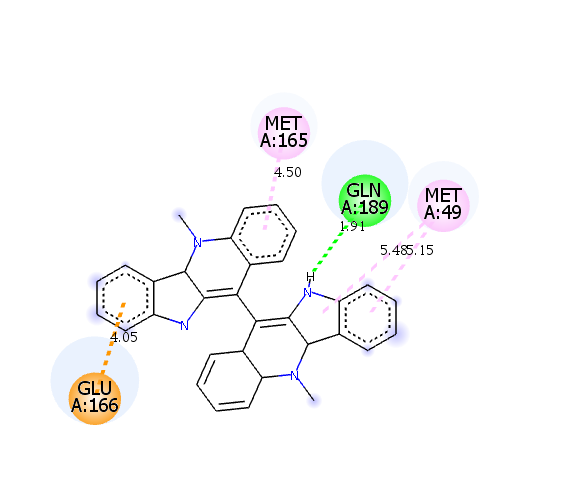

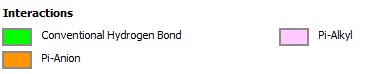


**A**

**B**

**
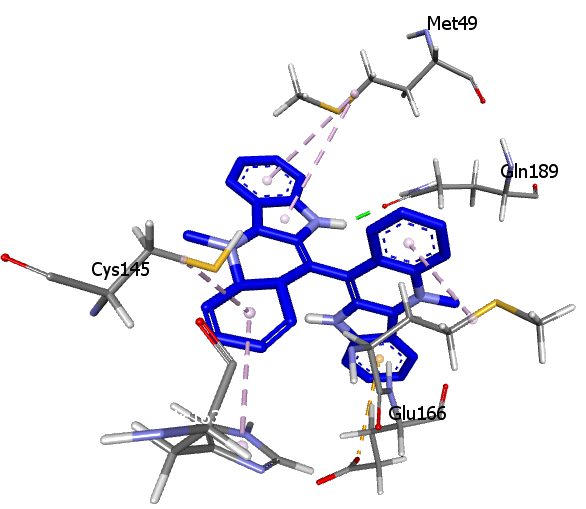
**

**Fig. S4.:**

(A) View of 3D interaction of cryptoquindoline with M^pro^ pocket residues (left with black labels) and 2D interactions colored by interaction type explained in legend (right). Cryptoquindoline exhibited a good binding affinity for M^pro^. From our result, a total of five (5) favorable interactions, which included a strong conventional hydrogen bond at a distance 3.55 Å, one pi- sulfur interaction with Cys145 amino acid residue, as well as three (3) pi-alkyl interactions with Met49 and Met165. The total of 3 hydrophobic interactions were seen in detail.

(B) View of 3D interaction of biscryptolepine with M^pro^ pocket residues (left with black labels) and 2D interactions colored by interaction type explained in legend (right). Biscryptolepine binding affinity of -8.8 kcal/mol from biscryptolepine, interaction with M^pro^ gave, there were a total of 5 favorable interactions; which included one conventional hydrogen bond at 1.86 Å, one pi-anion involved Glu166 residue, three (3) pi-alkyl interactions involved Met49 and Met165 residues, summing hydrophobic interactions to three (3).

**Table S1.:** Energetic contributions for free energy computation on selected protein ligand complexes used in molecular dynamics simulation

| **M^pro^** | ELE | VDW | GAS | PBSOL | PBTOT | GBSOL | GBTOT | TS | deltaPB | delatGB |
| --- | --- | --- | --- | --- | --- | --- | --- | --- | --- | --- |
| Cryptospirolepine | -1.05 | -44.9 | -45.96 | 16.34 | -29.61 | 9.12 | -36.83 | 13.96 | -15.65 | -22.87 |
| Cryptomisirine | -2.99 | -53.4 | -56.39 | 21.01 | -35.38 | 10.96 | -45.43 | 21.06 | -14.32 | -24.37 |
| Biscryptolepine | -2.65 | -49.15 | -51.8 | 19.9 | -31.9 | 11.42 | -40.38 | 19.22 | -12.68 | -21.16 |
| Cryptoquindoline | -2.03 | -43.86 | -45.88 | 15.87 | -30.02 | 9.15 | -36.73 | 21.59 | -8.43 | -15.14 |
| **RdRp** |  |  |  |  |  |  |  |  |  |  |
| Cryptomisirine | -10.54 | -53.39 | -63.93 | 24.46 | -39.47 | 17.85 | -46.08 | -14.07 | -53.54 | -60.15 |
| Cryptospirolepine | 0.72 | -49.45 | -48.72 | 17.75 | -30.98 | 8.23 | -40.49 | -13.96 | -44.94 | -54.45 |
| Cryptoquindoline | -2.39 | -49.64 | -52.03 | 20.15 | -31.88 | 9.38 | -42.65 | -13.03 | -44.91 | -55.68 |
| RemTP | -366.4 | -38.64 | -405 | 498.8 | 93.77 | 442.28 | 37.25 | -4.55 | 89.22 | 32.7 |
| **RdRpol** |  |  |  |  |  |  |  |  |  |  |
| Cryptospirolepine | -8.12 | -41.94 | -50.06 | 17.34 | -32.72 | 14.06 | -36 | 15.65 | -17.07 | -20.35 |
| Cryptomisirine | -10.8 | -44.36 | -55.15 | 23.45 | -31.7 | 18.77 | -36.38 | 19.46 | -12.24 | -16.92 |
| Cryptoquindoline | -1.01 | -43.92 | -44.93 | 15.7 | -29.23 | 10.02 | -34.91 | 24.25 | -4.98 | -10.66 |

Energy calculation includes MM/PB(GB)SA and consists of electrostatic energy as calculated by the MM force field (ELE), van der Waals contribution from MM (VDW), total gas phase energy (GAS), sum of non-polar and polar contributions to solvation (PBSOL/GBSOL). The final estimated binding free energy (deltaPB/ deltaGB) was calculated from the terms above (PBTOT/GBTOT) and entropy (TS), with unit kcal/mol.

**Table S2.**: ADMET profile predicted for the best four *Cryptolepis sanguinolenta* alkaloids and lopinavir

| **Property** | **Cryptomisrine** | | **Cryptoquindoline** | | **Cryptospirolepine** | | **Biscryptolepine** | | **Lopinavir** | |
| --- | --- | --- | --- | --- | --- | --- | --- | --- | --- | --- |
| **Physicochemical Property** | **Predicted Value** | **Probability** | **Predicted Value** | **Probability** | **Predicted Value** | **Probability** | **Predicted Value** | **Probability** | **Predicted Value** | **Probability** |
| Log S (Solubility) | -6.261 log mol/L (0.253 μg/ml) |  | -6.398 log mol/L (0.179 μg/ml) |  | -5.85 log mol/L (0.713 μg/ml) |  | -6.283 log mol/L (0.241 μg/ml) |  | -4.763lo mol/L (10.852 μg/ml) |  |
| LogD7.4 (Distribution Coefficient D) | 3.234 |  | 3.517 |  | 3.404 |  | 3.322 |  | 1.76 |  |
| Log P (Distribution Coefficient P) | 6.924 |  | 7.477 |  | 6.984 |  |  |  | 4.328 |  |
| **Absorption** |  |  |  |  |  |  |  |  |  |  |
| Papp (Caco-2 Permeability) | -5.06cm/s |  | -4.798 cm/s |  | -4.972 cm/s |  | -4.792 cm/s |  | -5.351 cm/s |  |
| Pgp- inhibitor | - | 0.443 | + | 0.524 | ++ | 0.715 | - | 0.304 | ++ | 0.819 |
| Pgp-substrate | --- | 0.068 | ++ | 0.757 | + | 0.681 | + | 0.626 | - | 0.417 |
| HIA (Human Intestinal Absorption) | ++ | 0.898 | ++ | 0.852 | ++ | 0.834 | +++ | 0.927 | --- | 0.187 |
| F (20% Bioavailability) | ++ | 0.714 | ++ | 0.752 | + | 0.693 | ++ | 0.706 | - | 0.336 |
| F (30% Bioavailability) | - | 0.452 | - | 0.474 | - | 0.386 | + | 0.676 | - | 0.352 |
| **Distribution** |  |  |  |  |  |  |  |  |  |  |
| PPB (Plasma Protein Binding) | 75.53% |  | 71.536% |  | 74.588% |  | 72.493% |  | 97.551% |  |
| VD (Volume distribution) | 0.291L/kg |  | 0.59L/kg |  | 0.78L/kg |  | 0.625 L/kg |  | -0.189 L/kg |  |
| BBB (Blood-Brain Barrier) | +++ | 0.918 | +++ | 0.95 | +++ | 0.959 | +++ | 0.988 | --- | 0.145 |
| **Metabolism** |  |  |  |  |  |  |  |  |  |  |
| P450 CYP1A2 inhibitor | ++ | 0.805 | +++ | 0.901 | --- | 0.24 | +++ | 0.976 | --- | 0.108 |
| P450 CYP1A2 Substrate | - | 0.344 | ++ | 0.734 | + | 0.664 | ++ | 0.828 | + | 0.504 |
| P450 CYP3A4 inhibitor | + | 0.5 | + | 0.541 | ++ | 0.89 | --- | 0.186 | ++ | 0.87 |
| P450 CYP3A4 Substrate | - | 0.306 | + | 0.629 | ++ | 0.72 | + | 0.634 | ++ | 0.83 |
| P450 CYP2C9 inhibitor | - | 0.328 | - | 0.424 | - | 0.464 | --- | 0.12 | + | 0.573 |
| P450 CYP2C9 substrate | + | 0.528 | + | 0.531 | + | 0.502 | + | 0.503 | --- | 0.211 |
| P450 CYP2C19 inhibitor | ++ | 0.715 | ++ | 0.717 | + | 0.632 | + | 0.5 | --- | 0.137 |
| P450 CYP2C19 substrate | --- | 0.298 | ++ | 0.701 | ++ | 0.754 | ++ | 0.745 | - | 0.373 |
| P450 CYP2D6 inhibitor | - | 0.407 | + | 0.551 | - | 0.488 | + | 0.576 | - | 0.449 |
| P450 CYP2D6 substrate | - | 0.313 | - | 0.435 | - | 0.377 | - | 0.41 | --- | 0.168 |
| **Elimination** |  |  |  |  |  |  |  |  |  |  |
| T_1/2_ (Half Life Time) | 2.525 h |  | 2.519 h |  | 2.442 h |  | 2.246 h |  | 1.863 h |  |
| CL (Clearance Rate) | 1.486 mL/min/kg |  | 1.527mL/min/kg |  | 1.520 mL/min  /kg |  | 1.588 mL/min  /kg |  | 1.364 mL/min/kg |  |
| **Toxicity** |  |  |  |  |  |  |  |  |  |  |
| hERG (heERG Blockers) | ++ | 0.78 | ++ | 0.787 | ++ | 0.822 | ++ | 0.764 | ++ | 0.77 |
| H-HT (Human Hepatotoxicity) | --- | 0.274 | --- | 0.138 | --- | 0.14 | --- | 0.11 | + | 0.692 |
| AMES (Ames mutagenicity) | + | 0.67 | ++ | 0.848 | - | 0.426 | - | 0.416 | --- | 0.16 |
| SkinSen(Skin Sensitization) | - | 0.358 | --- | 0.289 | --- | 0.29 | --- | 0.285 | --- | 0.212 |
| LD_50_ (LD_50_ of acute toxicity) | 2.763 –log mol/kg (796.516 mg/kg) |  | 2.868 –log mol/kg (607.842 mg/kg) |  | 3.436 –log mol/kg (184.902mg/kg) |  | 2.803 –log mol/kg (728.055 mg/kg) |  | 3.042 –log mol/kg (570.85mg/kg) |  |
| DILI(Drug Induced Liver Injury) | ++ | 0.844 | ++ | 0.852 | + | 0.698 | ++ | 0.712 | + | 0.556 |
| FDAMDO (Maximum Recommended daily dose) | + | 0.538 | - | 0.374 | - | 0.388 | + | 0.538 | - | 0.432 |

**Table S3**: ADMET profile predicted for Omeprazole and Ibuprofen.

| **Property** | **Omeprazole** | | **Ibuprofen** | |
| --- | --- | --- | --- | --- |
| **Physicochemical Property** | **Predicted Value** | **Probability** | **Predicted Value** | **Probability** |
| Log S (Solubility) | -3.975 log mol/L (36.589μg/ml) |  | -3.736 log mol/L (37.885 μg/ml) |  |
| LogD7.4 (Distribution Coefficient D) | 1.074 |  | 0.69 |  |
| Log P (Distribution Coefficient P) | 2.418 |  | 3.073 |  |
| **Absorption** |  |  |  |  |
| Papp (Caco-2 Permeability) | -4.228 cm/s |  | -4.379 cm/s |  |
| Pgp- inhibitor | + | 0.678 | --- | 0.089 |
| Pgp-substrate | --- | 0.009 | --- | 0.057 |
| HIA (Human Intestinal Absorption) | ++ | 0.751 | ++ | 0.857 |
| F (20% Bioavailability) | ++ | 0.812 | ++ | 0.816 |
| F (30% Bioavailability) | ++ | 0.763 | ++ | 0.769 |
| **Distribution** |  |  |  |  |
| PPB (Plasma Protein Binding) | 87.19% |  | 87.59% |  |
| VD (Volume distribution) | -0.017 L/kg |  | -1.299 L/kg |  |
| BBB (Blood-Brain Barrier) | --- | 0.043 | +++ | 0.991 |
| **Metabolism** |  |  |  |  |
| P450 CYP1A2 inhibitor | ++ | 0.827 | --- | 0.003 |
| P450 CYP1A2 Substrate | ++ | 0.777 | --- | 0.288 |
| P450 CYP3A4 inhibitor | +++ | 0.931 | --- | 0.004 |
| P450 CYP3A4 Substrate | +++ | 0.962 | --- | 0.148 |
| P450 CYP2C9 inhibitor | --- | 0.048 | --- | 0.01 |
| P450 CYP2C9 substrate | ++ | 0.891 | +++ | 0.989 |
| P450 CYP2C19 inhibitor | ++ | 0.879 | --- | 0.096 |
| P450 CYP2C19 substrate | +++ | 0.97 | ++ | 0.782 |
| P450 CYP2D6 inhibitor | - | 0.439 | --- | 0.219 |
| P450 CYP2D6 substrate | ++ | 0.713 | --- | 0.122 |
| **Elimination** |  |  |  |  |
| T_1/2_ (Half Life Time) | 1.801 h |  | 0.801 h |  |
| CL (Clearance Rate) | 1.577 mL/min/kg |  | 0.536 ml/min/kg |  |
| **Toxicity** |  |  |  |  |
| hERG (heERG Blockers) | ++ | 0.782 | --- | 0.284 |
| H-HT (Human Hepatotoxicity) | ++ | 0.844 | +++ | 0.934 |
| AMES (Ames mutagenicity) | - | 0.31 | --- | 0.028 |
| SkinSen(Skin Sensitization) |  | 0.194 | + | 0.613 |
| LD_50_ (LD_50_ of acute toxicity) | 2.291 -log mol/kg  (1767.472 mg/kg) |  | 1.907 –log mol/kg (2555.452 mg/kg) |  |
| DILI(Drug Induced Liver Injury) | ++ | 0.814 | + | 0.572 |
| FDAMDO (Maximum Recommended daily dose) | - | 0.312 | +++ | 0.936 |
